# Nuclear Envelope Membrane Protein 1 plays crucial and conserved roles in female meiosis

**DOI:** 10.21203/rs.3.rs-7159889/v1

**Published:** 2025-08-04

**Authors:** Bilal Ahmad Hakim, Yonit Tsatskis, Ling Zhang, Esther Choi, Ying Zhang, Didier Hodzic, Que Wu, MuYun Zhang, Maryam Pashei, Kyungwon Ha, Julie A. Brill, Miguel Angel Brieño-Enríquez, Andrea Jurisicova, Helen McNeill

**Affiliations:** 1 Department of Developmental Biology, Washington University School of Medicine, St. Louis, MO 63110, USA; 2 Cell & Systems Biology Program, The Hospital for Sick Children, Toronto, ON M5G 0A4, Canada; 3 Magee-Womens Research Institute, University of Pittsburgh, Pittsburgh, PA, USA; 4 Lunenfeld Tanenbaum Research Institute, Sinai Health Systems, Toronto, ON M5G 1X5, Canada; 5 Department of Obstetrics and Gynaecology and Physiology, University of Toronto, Toronto, ON M5G 1E2, Canada; 6 Department of Molecular Genetics, University of Toronto, Toronto, ON M5S 3K3, Canada

## Abstract

Female germ cells must preserve the integrity of their genome and generate genetic diversity via meiotic recombination. This challenging process, which occurs during fetal life, is error prone. Highly conserved checkpoint pathways detect errors in recombination and DNA damage, inducing the death of defective oocytes. Nuclear Envelope Membrane Protein (NEMP) homologs are highly conserved inner nuclear membrane proteins which are critical for fertility in flies, worms, fish and mice, and mechanically support the nuclear envelope. However, why NEMP homologs are required for fertility is still unclear. Using both *Drosophila* and mouse models, we establish here that loss of Nemp1 leads to activation of an ATM-CHK2 DNA damage pathway and results in massive loss of oocytes during fetal life. Chemical or genetic inactivation of the ATM-CHK2-p63 pathway reduces oocyte loss, demonstrating its importance upon loss of Nemp1. In the absence of Nemp1 meiotic progression is delayed and DNA damage is increased at zygonema and pachynema stages. Loss of Nemp1 also leads to defects in chromosome synapsis persisting through pachynema. We conclude that Nemp1 is needed for timely and precise execution of meiotic prophase and is crucial for accurate pairing and synapsis, oocyte developmental competence and survival.

## Introduction

Transmitting accurate genetic information to the next generation is critical for the maintenance of species. All the oocytes that a woman has for her entire reproductive lifetime are created during fetal life. Millions of germline cells are generated during early embryogenesis, and subsequently this pool is dramatically reduced, with more than 80% of oocytes dying during fetal life or shortly after birth^1–3^. Damage in oocytes can be devastating to the next generation, and canonical germline checkpoints are activated to ensure elimination of defective oocytes, including the DNA integrity sensing pathways^4,5^. Numerous reasons for this drastic reduction of oocytes have been proposed, including inability to repair DNA damage incurred during meiosis, defective chromosome pairing and synapsis, or the selective death of germ cells within cysts to enrich the oocytes with cytoplasmic components^6–10^. Most of this death is thought to occur via apoptosis, although autophagy and necroptosis have also been suggested to cull defective oocytes^11^.

A new player in the field of germ cell biology is the 5 transmembrane, inner nuclear envelope protein Nuclear Envelope Membrane Protein 1 (Nemp1)^12^. NEMP proteins are needed for fertility in flies, worms, fish and mice. Nemp1 is strongly expressed in oocytes within primordial follicles in adult mice, and loss of Nemp1 leads to dramatic reduction of primordial follicle number, with surviving oocytes having poor developmental competence. However, why oocytes are lost in Nemp1 mutants is unknown.

Here we show that loss of Nemp1 leads to activation of the ATM-CHK2 pathway, and that the loss of oocytes in flies and mice lacking Nemp1 can be rescued by inhibiting ATM-CHK2. We show that oocyte loss occurs during fetal stages and that loss of Nemp1 leads to meiotic defects, including failure in pairing, synapsis and recombination, providing an explanation for oocyte loss in Nemp1 mutants. These data place the Nemp1 family of nuclear envelope proteins as critical and conserved elements of meiosis and oocyte genome integrity.

## Results

### Loss of oocytes in *Nemp1*^*−/−*^ mutants begins in fetal life and is due to apoptosis

**Figure 1.**
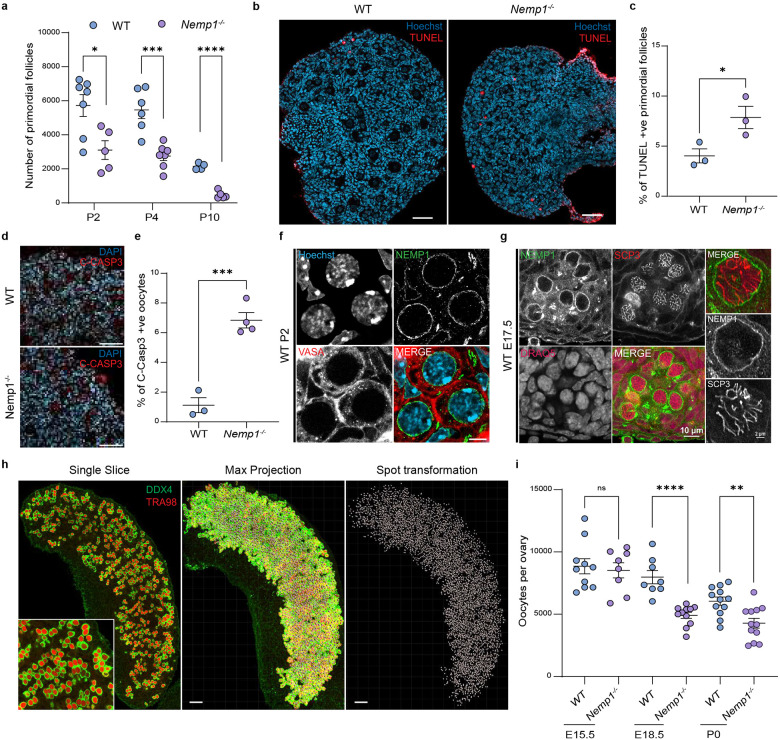
Loss of embryonic and postnatal oocytes in *Nemp1*^*−/−*^ mice occurs due to apoptosis. a) Follicle count showing a significant gradual loss of primordial follicles in *Nemp1*^*−/−*^ ovaries at postnatal day 2 (P2), P4 and P10. *P < 0.05, ***P < 0.001, ****P < 0.0001 (Unpaired t-test). Each dot represents the number of primordial follicles per ovary. b) Representative images showing normal primordial follicles and fewer TUNEL-positive (apoptosis marker) primordial follicle oocytes in P4 WT control ovaries, while an increased number of TUNEL-positive primordial follicle oocytes in P4 *Nemp1*^*−/−*^ ovaries (Scale Bars - 50 μm). c) Quantitative analysis of TUNEL-positive primordial follicles of P4 mice. *P < 0.05 (Unpaired t-test). Each dot represents number of TUNEL-positive primordial follicle oocytes per ovary. d) Activated caspase 3 (red), a marker of apoptosis, staining in WT vs *Nemp1*^*−/−*^ P2 ovarian sections showing increased cleaved caspase 3 expression in *Nemp1*^*−/−*^ sections (Scale Bars - 50 μm). e) Intensity analysis of cleaved caspase 3 in WT vs *Nemp1*^*−/−*^ ovaries at P2. ***P < 0.001 (Unpaired t-test). f) Nemp1 staining (green) in P2 WT VASA (red) positive oocytes (Scale Bars - 5 μm). g) Immunofluorescence labeling of Nemp1 (green) in embryonic 17.5 (E17.5) meiotic prophase SCP3 (red) positive oocytes. DNA is stained with DRAQ5 (Magenta). h) Representative images of 3D and germ cell spots analysis of whole-mount E15.5 WT ovary stained with germ cell markers, DDX4 (green) and TRA98 (red) (Scale Bars - 50 μm). i) Germ cell count shows a normal oocyte number in Nemp1 mutant embryonic 15.5 (E15.5) ovaries but a significant progressive oocyte loss in E18.5 and post-natal day 0 ovaries. ****P < 0.0001, **P < 0.01 (Unpaired t-test). Each dot represents the number of oocytes per ovary.

**Figure 2.**
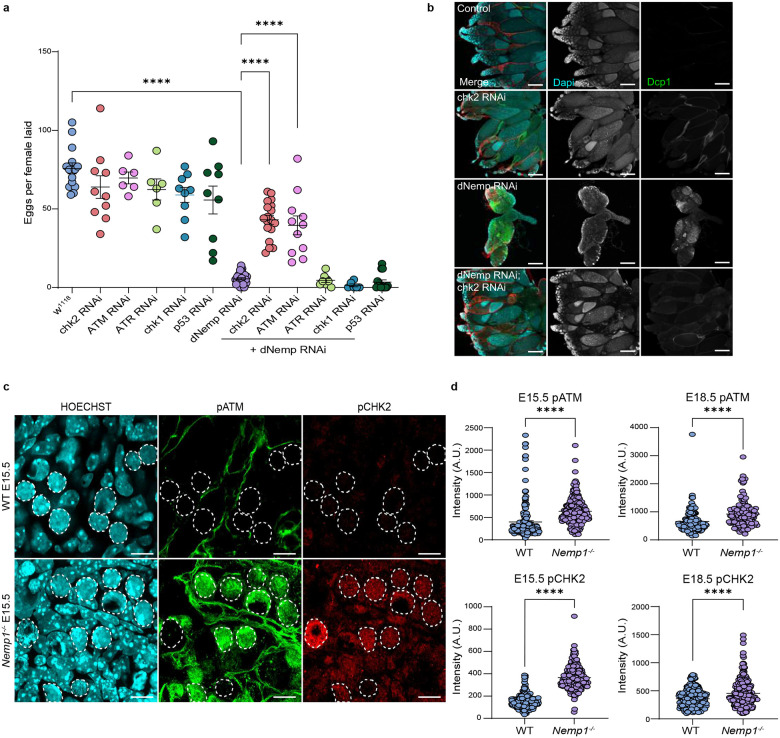
Activation of ATM and CHK2 causes germ cell loss in dNemp flies and *Nemp1*^*−/−*^ ovaries. a) The number of eggs laid by 3-day-old females shows rescue of egg laying in dNemp RNAi flies by reduction of ATM and Chk2 (DNA double-strand break pathway) but not by depletion of ATR and Chk1 (DNA single-strand break pathway). ****P < 0.0001 (one-way ANOVA, Tukey’s multiple comparison test). b) Representative confocal images of ovaries dissected from 3-day-old females showing ovary size rescue by reduction of Chk2 in dNemp flies (Scale Bars - 200 μm). c) Immunostaining of pATM and pCHK2 in E15.5 WT and *Nemp1*^*−/−*^ ovaries. Dashed circles show germ cells (Scale Bars - 10 μm). d) Fluorescence intensity analysis of pATM and pCHK2 in WT and *Nemp1*^*−/−*^ E15.5 and E18.5 germ cells showed a significant increase in these cell cycle checkpoints in *Nemp1*^*−/−*^ germ cells. ****P < 0.0001 (Mann-Whitney nonparametric test).

Previously we have shown that *Nemp1*^*−/−*^ females have a severely reduced primordial follicle pool at post-natal day 28 (P28)^12^. This loss of primordial follicles could be due to excessive death or due to an accelerated transition into the primary stage. We quantified primordial and primary follicle numbers in *Nemp1*^*−/−*^ mutants at P2, P4, and P10 (Fig 1a). Histological analysis showed no significant difference in the number of primary follicles at P10 (Fig 1a), suggesting recruitment is not affected and that excessive death is the cause of primordial follicle loss. Examination of P4 *Nemp1*^*−/−*^ ovaries also showed ~50% reduction of primordial follicles (P value < 0.01) (Fig 1a). TUNEL staining of P4 ovaries revealed increased TUNEL-positive primordial follicles in *Nemp1*^*−/−*^ ovaries (P Value < 0.05) (Fig 2b and 2c). Activated caspase-3 expression, another marker of apoptosis, was also increased in *Nemp1*^*−/−*^ P2 ovaries (P Value < 0.001) (Fig. 2d and 2e). These data indicate there is increased apoptosis in germ cells in the absence of Nemp1.

To determine if Nemp1 could function earlier in development, we examined the expression of Nemp1 in fetal and neonatal ovaries. Immunofluorescence revealed Nemp1 is strongly expressed and localized on the nuclear envelope of oocytes at embryonic day 17.5 (E17.5) (Fig 1f) and maintained during the formation of follicles (Fig 1g).

The increased apoptosis in *Nemp1*^*−/−*^ neonatal ovaries and the prominent expression of Nemp1 in E17.5 oogonia led us to examine oocyte number and quality in fetal ovaries. We used wholemount analysis of intact fetal ovaries with the Clear, Unobstructed Brain/Body Imaging Cocktails and Computational analysis (CUBIC) method to clear ovarian tissues and reconstruct three-dimensional (3D) fluorescence images with the germ cell markers TRA98 and DDX4 (Fig 1h). We analyzed E15.5, E18.5, and P0 ovaries from wildtype and *Nemp1*^*−/−*^ animals. At E15.5, oocyte number in *Nemp1*^*−/−*^ ovaries was normal, but by E18.5 there was a significant loss of oocytes (reduction by ~39%; P value < 0.001). Reduced oocyte number was also observed at P0 in *Nemp1*^*−/−*^ ovaries (P Value < 0.05) (Fig 1i). Taken together, these data indicate that Nemp1 is required to sustain oocyte viability in fetal stages and maintain the ovarian reserve postnatally.

### ATM and CHK2 activation triggers oocyte loss in *dNemp* mutant flies

To understand the mechanism by which Nemp1 deficiency triggers oocyte loss, we turned to *Drosophila*, as flies are amenable to rapid genetic analyses. *dNemp* flies are viable but sterile, with significantly reduced ovary size and severe germ cell loss^12^. Emerin mutants also lack oocytes^13^, and oocyte loss can be prevented by inactivation of the DNA damage response kinases, ATR and Chk2^14^. Because dNemp binds *Drosophila* Emerin^12^, we tested if oocyte loss caused by knockdown of *dNemp* could also be rescued by inhibition of the DNA damage response (DDR) pathway. To investigate the role of the DDR in the loss of oocytes in *dNemp* mutants, we used RNAi knockdown of DDR pathway components ATM (*tefu*), ATR (*mei-41*), Chk1 (*grapes*), Chk2 (*loki, mnk*), and p53. As previously reported^12^, driving *dNemp* RNAi in the germline with Nanos Gal4 causes loss of oocytes, leading to reduced egg laying (Fig 2a and 2b). Importantly, loss of egg laying and reduced ovary size in *dNemp* RNAi flies was significantly rescued by germline knockdown of the kinases ATM (P Value < 0.0001) and Chk2 (P Value < 0.0001) (Fig 2a, b and S2). We also examined the effects of loss of the single stranded DDR kinases ATR and Chk1; however, we found no rescue of egg laying or ovary size by germline knockdown of these genes in *dNemp* mutant flies. These data imply that loss of dNemp in germ cells triggers oocyte loss by activation of ATM-CHK2 pathway, pointing to double stranded DNA damage as a potential cause of oocyte loss in *dNemp* mutants.

Reduced oocyte number in *dNemp* mutants is likely due to apoptosis as there is increased expression of Dcp-1, an effector caspase (Fig 2b). The increased Dcp-1 was eliminated in *dNemp/Chk2* double mutants, indicating CHK2 activation drives this apoptosis (Fig 2b). We also performed double knockdowns of dNemp with initiator caspases (Dronc, Dredd and Strica) and executioner caspases (Drice, Dcp-1, Decay and Damm). Inhibition of Drice (homolog of Caspase-3) in *dNemp* flies rescued ovary size, but egg laying only slightly increased (Fig S3a and S3b). Knockdown of other caspases did not show any rescue. Knockdown of p53 resulted in a small rescue in ovary size of *dNemp* mutants (Fig S2) but had no effect on egg laying.

### ATM and CHK2 are activated in *Nemp1* mutant mouse oocytes

We next asked whether ATM and CHK2 checkpoints are also responsible for oocyte loss in *Nemp1*^*−/−*^ mice. To assess activation of ATM and CHK2, we immunostained E15.5 and E18.5 fetal and day 2 postnatal ovaries for ATM phosphorylated at serine-1981 and CHK2 phosphorylated at threonine-68^15,16^. Notably, both pATM and pCHK2 showed increased expression in *Nemp1* mutants (P Value < 0.0001) (Fig 2c, 2d, S4, S5, S6c and S6d), suggesting loss of Nemp1 activates a conserved DDR pathway for germ cell elimination.

Formation of primordial follicles, which starts shortly before birth and continues until ~day 4 postpartum, is also a period of selective germ cell death. Immunostaining of P2 *Nemp1*^*−/−*^ ovaries revealed elevated expression of the DNA damage marker γH2AX (P Value < 0.0001) (Fig S6a and S6b). TAp63a (hereafter p63), a key transcription factor responsible for eliminating oocytes with unrepaired DSBs, was also elevated in *Nemp1*^*−/−*^ ovaries (P Value < 0.0001) (Fig S6a and S6b). pATM levels were also increased in Nemp1 mutant ovaries (P Value < 0.0001) (Fig S6c and S6d) indicating that ATM signaling and DNA damage persists.

### Pharmacological inhibition of ATM and CHK2 rescues oocyte loss in Nemp1 mutants

**Figure 3.**
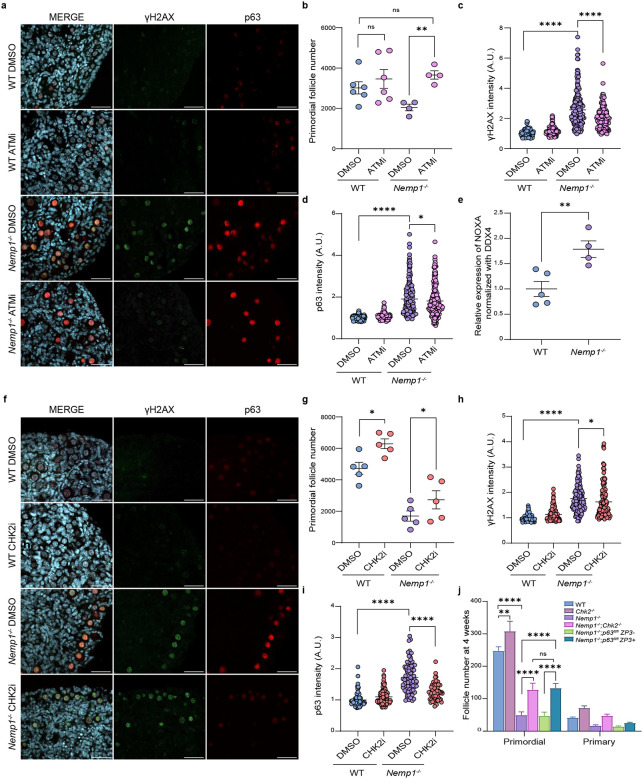
Inactivation of ATM, CHK2 and p63 pathway rescues primordial follicle loss in *Nemp1*^*−/−*^ mice. a) Representative immunofluorescence of solvent and ATM inhibitor treated P4 WT and *Nemp1*^*−/−*^ ovaries after 2 days of *in vitro* culture (Scale Bars - 50 μm). b) Follicle quantification, c) γH2ax, and d) p63 fluorescence intensity of solvent and ATM inhibitor treated WT and *Nemp1*^*−/−*^ ovaries showing ATM inhibitor rescue partial primordial follicle number and reduce DNA damage in *Nemp1*^*−/−*^ primordial follicle oocytes. *P < 0.05, **P < 0.01, ****P < 0.0001 (Paired t-test and Wilcoxon nonparametric test). e) Quantification of relative mRNA expression of NOXA in P2 WT and *Nemp1*^*−/−*^ ovaries by real-time PCR. **P < 0.01 (Unpaired t-test). f) Representative immunostaining of solvent and CHK2 inhibitor treated P4 WT and *Nemp1*^*−/−*^ ovaries after 2 days of *in vitro* culture (Scale Bars - 50 μm). g) Follicle quantification, h) γH2ax, and i) p63 fluorescence intensity of solvent and CHK2 inhibitor treated WT and *Nemp1*^*−/−*^ ovaries showing a partial rescue of primordial follicle number and p63 reduction in CHK2 inhibitor treated *Nemp1*^*−/−*^ ovaries compared with solvent treated *Nemp1*^*−/−*^ ovaries. *P < 0.05, ****P < 0.0001 (Paired t-test and Wilcoxon nonparametric test). j) Follicle count of 4-week-old control (WT), *Chk2*^*−/−*^, *Nemp1*^*−/−*^, *Nemp1*^*−/−*^;*Chk2*^*−/−*^, and *Nemp1*^*−/−*^;*p63*^*fl/fl*^ *ZP3+* mice ovaries show deletion of Chk2 and p63 rescues primordial follicle count in *Nemp1*^*−/−*^ animals. ****P < 0.0001 (two-way ANOVA, Tukey’s multiple comparison test).

To assess the role of ATM in *Nemp1*^*−/−*^ oocyte loss in mice, we cultured WT and *Nemp1*^*−/−*^ neonatal ovaries with an ATM inhibitor (KU55933; labelled ATMi). Pharmacological inhibition of ATM prevented germ cell loss (P Value < 0.01) (Fig 3b), as follicle number in ATMi-treated ovaries was comparable to that of WT ovaries (Fig 3b). As anticipated, γH2AX was reduced in ATMi-treated *Nemp1*^*−/−*^ ovaries (P Value < 0.0001) (Fig 3a and 3c). Expression of p63α was also reduced upon ATMi treatment (P Value < 0.05) (Fig 3a and 3d).

CHK2i treatment also resulted in rescue of primordial follicle number in *Nemp1*^*−/−*^ ovaries (P Value < 0.05) (Fig 3g). γH2AX also decreased in CHK2i-treated Nemp1 deficient ovaries (P Value < 0.05) (Fig 3f and 3h). p63 is a well-established CHK2 target in oocytes^4^, which activates cell death, and is responsible for elimination of oocytes in primordial follicles with DSB DNA breaks. p63 expression was strongly reduced in the CHK2i-treated *Nemp1*^*−/−*^ ovaries (P Value < 0.0001) (Fig 3f and 3i). These data indicate that Chk2 is activated in *Nemp1*^*−/−*^ and that inhibition of Chk2 or its target p63 can rescue the loss of oocytes. The p63 transcriptional targets NOXA and PUMA are proapoptotic genes critical for DNA damage-induced oocyte apoptosis^17^. *Nemp1*^*−/−*^ P2 ovaries showed elevated expression of NOXA (P Value < 0.01) (Fig 3e); however, PUMA transcript and p53 protein were undetectable.

### Genetic inactivation of CHK2 and its target p63 prevents oocyte loss in Nemp1 mutant mice

Our data indicate that pharmacological inhibition of ATM or CHK2 can suppress oocyte loss in *Nemp1* mutants. However, inhibitors can have off-target effects. Therefore, we generated *Nemp1 CHK2* double knockouts (*Nemp1*^*−/−*^;*Chk2*^*−/−*^). Importantly, genetic ablation of CHK2 in Nemp1 mutants partially rescued primordial follicle number (P Value < 0.0001) (Fig 3j).

We also examined the functional requirement of the Chk2 target p63 using oocyte specific ZP3-cre and conditional alleles of p63 (*Nemp1*^*−/−*^;*TAp63*^*fl/fl*^ *ZP3-Cre*). Inactivation of *p63* in *Nemp1*^*−/−*^ background partially rescued the primordial follicle pool (P Value < 0.0001) (Fig 3j). Together, these results indicate that loss of Nemp1 activates a conserved ATM-CHK2-p63 checkpoint to ensure the elimination of damaged oocytes.

Nemp1 deficient females ovulate oocytes with spindle defects^12^. DNA damage in oocytes is known to activate the spindle assembly checkpoint, preventing meiotic completion^18^. To determine if loss of Chk2 can rescue the spindle defects of *Nemp1* mutants, we performed acetylated tubulin staining on ovulated MII oocytes. CHK2 inactivation partially inhibited Nemp1 spindle defects and chromosome misalignment (Fig S7a and S7b). In contrast, loss of p63 did not rescue these defects (Fig S7a and S7b), but did rescue DNA damage, as evidenced by rescue of comet length (Fig S7c and S7d), However, neither loss of CHK2 nor p63 restored fertility in *Nemp1*^*−/−*^ females (Fig S7e), suggesting Nemp1 may have other functions during later oocyte development or early embryonic development.

### *Nemp1*^*−/−*^ fetal oocytes show increased DNA damage

To understand if elimination of oocytes in *Nemp1*^*−/−*^ mice is related to defects in pairing, synapsis and recombination checkpoint activation, we performed chromosome spreads in oocytes at E15.5 and E17.5. Meiotic prophase progression is defined by two features: the synapsis of homologous chromosomes and initiation of meiotic recombination by DNA double-strand break formation^19,20^. We defined meiotic prophase I substages using the cytological appearance of the synaptonemal complex proteins (SYCP1 and SYCP3)^21,22^. Leptonema is the first substage and follows pre-meiotic DNA replication that results in the sister chromatids being physically linked together via the cohesin complex^23^. Later, during zygonema, homologous chromosomes pair and begin to synapse as the central element proteins of the synaptonemal complex promote this process. Finally, once the homologous chromosomes are completely condensed and synapsed, the cell in pachynema proceeds to the last steps of repairing the programmed double stranded breaks (DSBs)^20^.

**Figure 4.**
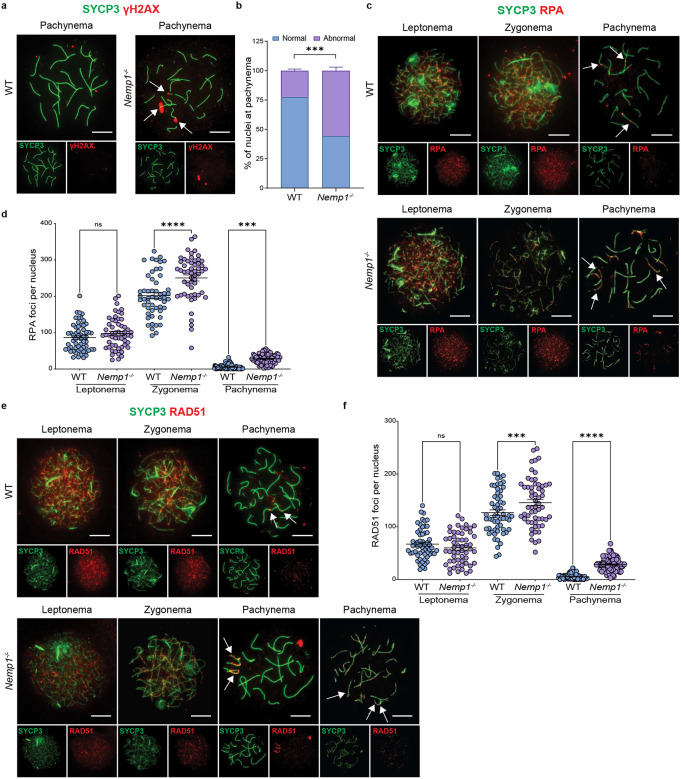
Nemp1 loss leads to persistent DNA damage in embryonic oocytes. a) Representative images of WT and *Nemp1*^*−/−*^ pachynema oocyte spreads at E17.5 showing persistent γH2AX (red) staining in *Nemp1*^*−/−*^ oocytes indicating DNA Damage. Oocytes are immunostained for SYCP3 (green) and γH2AX (red) (Scale Bars - 10 μm). b) Proportion of oocytes with persistent γH2AX (abnormal) in WT and *Nemp1*^*−/−*^ E17.5 pachynema spreads. ***P < 0.001 (Unpaired t-test). c) Representative images of WT and *Nemp1*^*−/−*^ E17.5 oocytes stained with SYCP3 (green) and RPA (red) showing leptonema, zygonema, and pachynema stages (Scale Bars - 10 μm). d) Quantification of RPA foci from three meiotic stages. ****P < 0.0001, ***P < 0.001 (one-way ANOVA, Sidak’s multiple comparison test). e) Representative images of WT and *Nemp1*^*−/−*^ E17.5 oocytes stained with SYCP3 (green) and RAD51 (red) showing leptonema, zygonema, and pachynema stages (Scale Bars - 10 μm). f) Quantification of RAD51 foci from three meiotic stages. ***P < 0.001, ****P < 0.0001 (one-way ANOVA, Sidak’s multiple comparison test).

We first analyzed DNA DSBs formation using γH2AX. As expected, in wildtype oocytes there is strong γH2AX during leptotene and zygotene stages, which decreases to ~ 20% as DSB breaks become resolved in pachytene. However, in *Nemp1*^*−/−*^, ~54% of oocytes at late pachytene showed persistent γH2AX signal (P Value < 0.001) (Fig 4a and 4b). γH2AX staining was associated with both synapsed and asynapsed bivalents. Staining *Nemp1*^*−/−*^ oocytes with CREST showed variable centromere number (Fig S8), suggesting that loss of Nemp1 results in aneuploidy.

To understand if the repair of chromosomes in *Nemp1*^*−/−*^ oocytes proceeds normally, we evaluated the spatial-temporal distribution of repair proteins. First, we evaluated the presence of single stranded DNA from leptonema to pachynema, using replication protein A (RPA); we observed that the RPA foci number was not significantly different at leptonema, however at zygonema *Nemp1*^*−/−*^ oocytes showed a significant increase of RPA foci per nucleus, compared to controls (Fig 4c and 4d). At pachynema, RPA foci were reduced in both Nemp1 mutants and controls; however, *Nemp1*^*−/−*^ retained more RPA foci (Control 5.48± 0.64 vs *Nemp1*^*−/−*^ 28.09±1.37) (Fig 4c and 4d). Next, we analyzed if Nemp1 loss can affect single strand invasion during the DNA damage and repair pathway of meiotic prophase I, for that purpose we analyzed RAD51. As with RPA, no differences were observed at leptonema. However, at zygonema and pachynema, Nemp1 mutants showed a significant increase of RAD51 foci (zygonema: control 126.8±5.33 vs *Nemp1*^*−/−*^ 145.7±6.03; pachynema: control 5.43±0.48 vs Nemp1 mutant 28.48±1.18) (Fig 4e and 4f). Taking together these data indicate that loss of Nemp1 leads to persistent DNA damage.

### Nemp1 mutants are defective in synapsis and recombination

**Figure 5.**
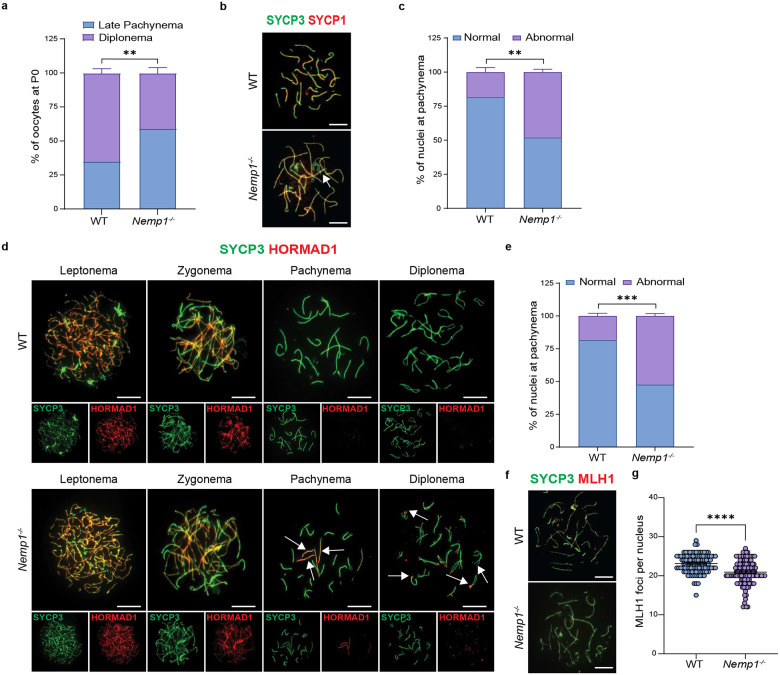
Nemp1 loss leads to defects in synapsis and recombination. a) Percentage of oocyte spreads in P0 WT and *Nemp1*^*−/−*^ ovaries suggesting a delay in Meiosis I progression. **P < 0.01 (Unpaired t-test). b) Examples of fully synapsed and asynapsed oocytes, where the white arrow shows the site of asynapsis (Scale Bars - 10μm). c) Quantification of asynapsis. **P < 0.01 (Unpaired t-test). d) Representative images of WT and *Nemp1*^*−/−*^ E17.5 oocytes stained with SYCP3 (green) and Hormad1 (red) showing leptonema, zygonema, pachynema, and diplonema stages (Scale Bars - 10μm). e) Percentage of nuclei at pachynema showing retention of Hormad1 staining in WT and *Nemp1*^*−/−*^ oocytes at E17.5. ***P < 0.001 (Unpaired t-test). White arrows show Hormad1 retention in *Nemp1*^*−/−*^ oocytes. f) Representative images of WT and *Nemp1*^*−/−*^ E17.5 pachynema oocyte spread stained with SYCP3 (green) and MLH1 (red) (Scale Bars −10μm). g) Quantification of MLH1 foci at pachynema. ****P < 0.0001 (Unpaired t-test).

Analysis of chromosome spreads at P0 showed that a majority of *Nemp1*^*−/−*^ oocytes (~60%) were in late-pachytene stage compared to only 35% in wildtype (P Value < 0.01) (Fig 5a) suggesting a delay in meiosis I progression. We next evaluated the progression of meiotic synapsis in *Nemp1*^*−/−*^. We stained nuclear spreads using antibodies against synaptonemal complex proteins SYCP3 and SYCP1, which recognize synapsing chromosomes, and antibodies against HORMAD1, which is retained on unsynapsed regions in prophase I oocytes. Analysis of embryonic meiotic spreads revealed that ~50% of *Nemp1*^*−/−*^ oocytes display asynapsis compared to ~20 % in the WT oocytes. We observed defects in synapsis characterized by both retention of HORMAD1 and lack of SYCP1 on chromosomes of pachytene-like cells (Fig 5b, 5c, 5d and 5e). We next evaluated formation of crossovers using MLH1 (MutL protein homolog 1). The overall number of MLH1 foci in controls was (23.13 ± 0.22), however in *Nemp1*^*−/−*^ oocytes, MLH1 average foci number decreased slightly (20.89 ± 0.28 foci) (Fig 5f and 5g). Interestingly however, the numbers of MLH1 foci varied in *Nemp1*^*−/−*^ cells: some completely lacked MLH1 foci (13% of the cells) while others had normal numbers of MLH1 foci (18%). Taken together these data indicate that Nemp1 has a crucial role in meiotic prophase, and absence of Nemp1 leads to defects in pairing, synapsis and repair, providing an explanation for the loss of oocytes.

## Discussion

**Figure 6.**
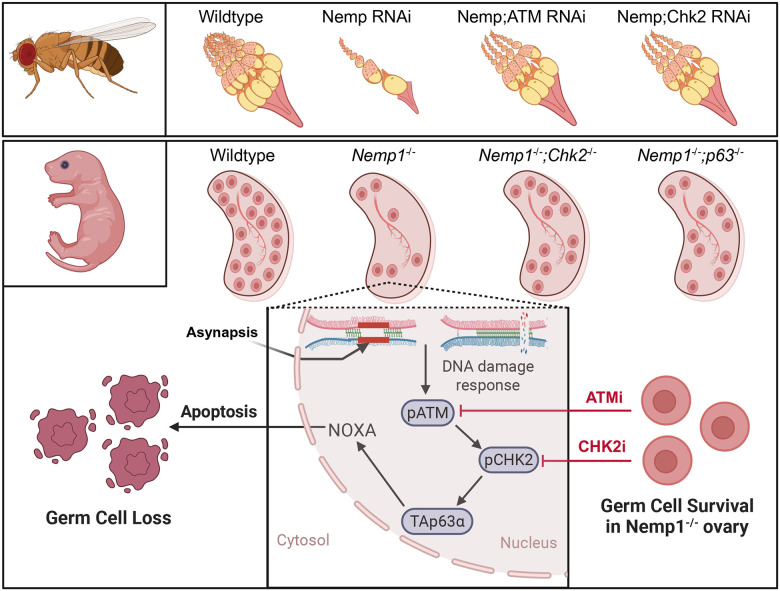
Graphical summary of Nemp1 deficiency triggered oocyte loss. Loss of Nemp1 in oocytes leads to excessive DNA damage, which activates the ATM-mediated CHK2 checkpoint in both flies and mice. This activation further triggers p63, resulting in NOXA-dependent apoptosis. Inhibition of ATM or CHK2, as well as genetic inactivation of CHK2 or p63, prevents oocyte loss in Nemp1 mutant ovaries. This demonstrates that the loss of Nemp1 alone is sufficient to activate a conserved checkpoint pathway, emphasizing its crucial role in the nuclear envelope of germ cells for maintaining genome integrity.

Our studies have identified a new, evolutionarily conserved player in female meiosis, the nuclear envelope membrane protein Nemp1 (Fig 6). We show here that Nemp1 is required for chromosomal synapsis and recombination, and that loss of Nemp1 activates an ATM-CHK2 DNA Damage Response (DDR). Importantly, inhibition of ATM/CHK2 can rescue oocyte loss in *dNemp* flies and *Nemp1*^−/−^ mice.

The canonical ATM-Chk2 checkpoint pathway is mainly activated by DNA double-strand breaks (DSBs), whereas the ATR-Chk1 checkpoint pathway responds to DNA replication defects and other forms of DNA damage^24^. In *Nemp1*^−/−^ germ cells, activation of ATM and CHK2 at E15.5 was followed by dramatic loss of oocytes at E18.5. Oocytes rapidly undergo meiotic events at this stage, and any perturbation during this period can lead to massive oocyte death via the DDR pathway^4,5,7–9,25^. *Nemp1*^−/−^ perinatal oocytes possess elevated DNA damage as marked by γH2aX staining. Inhibition of ATM in *Nemp1*^*−/−*^ cultured ovaries *in vitro* reduced DNA damage and prevented primordial follicle loss.

CHK2, the downstream effector of ATM, is activated in the presence of unrepaired DSBs and plays a crucial role in oocyte death due to persistent DSBs or asynapsis of homologous chromosome^4,7^. Genetic and pharmacological inhibition of CHK2 or p63 rescued primordial follicles but did not restore fertility of *Nemp1*^*−/−*^ mutants. This suggests that Nemp1 has additional functions later in development, likely by contributing to chromatin mediated oocyte developmental competence.

Meiotic defects detected by CHK2 in oocytes trigger activation of the downstream p53/TAp63 pathway, followed by PUMA/NOXA/BAX-dependent apoptosis^4,7,17^. The increased DNA damage in *Nemp1* oocytes is reflected by increased number of Rad51 and RPA foci in Nemp1 oocytes, as well as increased p63 and *NOXA* expression.

We observed a range of chromosomal abnormalities in *Nemp1*^*−/−*^ oocytes during meiotic prophase I, including incomplete synapsis of homologous chromosomes, defective recombination, persistent γH2AX hyperphosphorylation and delay in meiotic progression. In mice, oocyte elimination is regulated by several quality control checkpoints. The synapsis checkpoint eliminates oocytes with widespread asynapsis, functioning even in the absence of programmed DSBs, as observed in *Spo11*^*−/−*^ females^25–28^. Meiotic silencing of unsynapsed chromosomes inactivates genes on these chromosomes, causing oocyte elimination when a small number of chromosomes fail to synapse^29^. These processes involve Hormad1 and Hormad2 proteins, which we show persist in *Nemp1*^*−/−*^ oocytes^7,26,27,30,31^. Oocytes with faulty recombination repair, such as *Dmc1*^*−/−*^ or *Msh5*^*−/−*^, also show chromosome asynapsis and DNA damage, likely leading to elimination through the combined action of the synapsis and DNA-damage checkpoints^17,32–35^.

Why does loss of Nemp1 lead to synapsis and pairing defects, DNA damage and oocyte death? Nemp1 is an inner nuclear membrane protein, and its absence leads to a softer nuclear envelope^12^. This softness may hamper telomere-led chromosome movements that occur at the nuclear envelope, impacting pairing^36,37^. In addition, the pull exerted on the chromosome ends through the nuclear envelope is thought to reinforce cues establishment of the synaptonemal complex between homologous chromosomes and block nonhomologous synapsis, a key requirement for cross-over formation^38^. Chromosome movements also have a role in DNA repair by promoting the use of homologous recombination rather than the error-prone nonhomologous end joining pathway to repair DNA damage and DSBs that form during meiotic prophase. We have previously shown that Nemp1 supports nuclear envelope mechanical stiffness in oocytes, and our recent studies indicate that Nemp1 biochemically interacts with Nesprins (Ganguly et al, in submission). Nesprins have critical roles in the bouquet stage of meiosis, providing the mechanical linkage needed for telomere movement and clustering. We hypothesize that Nemp1 acting with Nesprin is needed for the normal pairing and synapsis that occurs during the bouquet stage, and that defects in this process lead to unresolved DNA damage due to lack of proper synapsis/repair. Taken together, these data reveal Nemp1 as a novel, critically important regulator of meiosis, and that Nemp1 loss leads to DNA damage, asynapsis, and recombination defects, thereby severely compromising genome integrity.

## Material and Methods

### *Drosophila melanogaster* lines

WT flies are *w*^*1118*^ unless otherwise noted. RNA interference (RNAi) stocks for expression of double-stranded RNA under control of GAL4/UAS were obtained from Bloomington Drosophila Stock Center (BDSC); *dNemp* BDSC_57475, *chk2* BDSC_35152, *ATM* BDSC_44417, *ATR* BDSC_35371, *chk1* BDSC_62155, *p53* BDSC_41638, *Dcp1* BDSC_38315, *Decay* BDSC_65879, *Dredd* BDSC_34070, *Drice* BDSC_32403, *Damm* BDSC_63622, *Strica* BDSC_54059, *Dronc* BDSC_32963 and were driven using Nanos *Gal4*-VP16.

### Mice

C57BL/6J mice were purchased from Jackson Laboratory. Nemp1 mutant mice (*Nemp1*^em#(TCP)McNeill^) were generated as previously described^12^. Chek2 mutant mice (*Chek2*^*tm1b(EUCOMM)Hmgu*^) were obtained from Ewelina Bolcun-Filas and were genotyped as previously described^39^. Nemp1^−/−^/Check2 KO mice were generated by crossing heterozygous Nemp1 females with homozygous Chek2 male mice. Conditional inactivation of TAp63 (Trp63^tm1Elrf^) on Nemp1 deficient background was achieved by introduction of oocytes specific Cre line (Zp3-cre^,3Mrt^). This Cre line is expressed in oocytes of primordial follicles with activity detected as early as day 17.5 dpc^40^. For embryonic experiments, females were set up in breeding and examined for copulation plugs daily. The plugging day was considered 0.5 days post coitum (dpc). Embryos were sacrificed at 15.5, 17.5, and 18.5 dpc.

### Immunostaining - adult *Drosophila* ovaries

3-day old adult *Drosophila* ovaries were dissected in PBS, fixed in 4% paraformaldehyde for 30 minutes, washed with PBT (0.3% Triton X-100 in PBS), blocked with PBT-BSA (PBT with 0.5% bovine serum albumin), incubated with Dcp1 Rabbit primary antibody (NEB Cat #9578S) in PBT-BSA at 4 C overnight, washed with PBT, incubated with FITC Rabbit secondary antibody (Jackson ImmunoResearch Cat # 711-095-152) in PBT-BSA for 1 hr along with phalloidin Rhodamine (ThermoFisher Cat #R415), washed with PBT and mounted in fresh Vectashield with DAPI (VectorLabs Cat #H-1200–10). Imaging was done with a Nikon A1R confocal microscope. Images represent individual optical sections.

### Immunofluorescence of mouse ovaries

Whole ovaries harvested from embryos and neonates were fixed in 4% paraformaldehyde for 3 hours. For frozen sections, ovaries were allowed to equilibrate overnight in 30% sucrose/PBS at 4°C. Ovary sections (20 μm) were collected on Superfrost Plus microscope slides (Fisher Scientific) from optical cutting temperature (OCT) embedded blocks using a cryostat (CM 1850, Leica). For paraffin sections ovaries were processed through routine embedding and sectioned at 5uM. Before immunostaining, antigen retrieval in sodium citrate buffer was performed by heating sections. Afterward, sections were rinsed in PBS, permeabilized, and blocked with 10% donkey serum/0.5% Triton X-100 in PBS for 1 hour followed by an overnight incubation with primary antibodies at 4°C. Hoechst/DAPI and appropriate Alexa 488/594/647–conjugated secondary antibodies (Thermo Fisher Scientific) were added to the blocking buffer for 2 hours after rinsing in PBS. Finally, the sections were mounted with Dako fluorescence mounting medium (Agilent Technologies), and images were taken on a Ti2 inverted confocal laser microscope (Nikon) with the NIS-Elements software using a 60× objective. Histomorphometric assessment of ovarian reserve at various timepoints was performed by systematic follicle counting of serial sections covering the entire ovary as previously described^40^.

### TUNEL immunofluorescence on cryosections

TUNEL labeling was performed on 20 μm cryosections to detect cell death with a situ Cell Death Detection Kit (Roche, Cat #12156792910). Fixed ovary cryosections were rinsed in PBS and permeabilized with PBT (0.5% Triton X-100 in PBS) for 20 min, again rinsed in PBS, incubated with 50 ul of TUNEL reaction mixture with enzyme solution in a humid chamber for 1 hour at 37°C. After rinsing the section 3x with PBS, nuclei were stained with Hoechst for 20 min, rinsed again 3x in PBS, and mounted with Dako fluorescence mounting medium (Agilent Technologies). Cryosections digested with DNase I (Invitrogen) served as positive control whereas negative control sections were incubated without the TUNEL enzyme solution.

### Preparation and immunofluorescence of oocyte spreads

Female meiotic surface spreads of prophase I oocytes were prepared from two ovaries of each mouse as described previously^28^. Briefly, after dissection, ovaries were placed in 20ul hypotonic extraction buffer containing 30mM Tris-HCl, 17 mM Trisodium citrate dihydrate, 5 mM EDTA, 50 mM sucrose, 0.5 mM PMSF, 0.5 mM DTT, 1x Protease Inhibitor Cocktail for 15 min at room temperature. Further ovaries were placed in 100 mM sucrose in 5 mM sodium borate buffer pH 8.5 and punctured by 29-gauge syringe needles to release oocytes. The cell suspension was incubated with 65 mM sucrose in 5 mM sodium borate buffer pH 8.5 for 3 min. The cell suspension was distributed into four 20 μL fixative drops (1% paraformaldehyde, 0.15% Triton X-100, 1 mM borate buffer, pH 9.2) on two glass slides enclosed by a hydrophobic barrier. Slides were incubated for 45 min in a humid chamber and then air dried. Further slides were washed in 0.4% Photo-Flo 200 solution, rinsed with distilled water, air dried again, and stored at −80°C until staining. For immunostaining, slides were brought to RT and washed with 10% blocking buffer (3%BSA/10% goat serum/0.05% Tween-20) in PBS for 10 min. Slides were blocked for 1 hour with blocking buffer followed by overnight primary antibodies incubation at 4°C. Secondary antibody incubation was carried out for 1.5 hours at RT. After washing, slides were mounted with Vectashield plus Antifade mounting medium with DAPI (VectorLabs Cat #H-2000–10).

### Whole mount staining of ovaries and optical clearing

Ovaries were harvested and fixed immediately in 4% PFA for 24 hours at RT, and dehydrated in 30% sucrose for 12 hours at RT. Ovaries with the same genotype were transferred into a bottle containing a blocking solution (10% donkey serum/1% Triton X-100) in PBS and incubated overnight. Next, 1ug primary antibodies were added to the bottle and incubated for 24 hours at 37°C. Ovaries were washed 5x in 2 ml PBS for 30 min each at 37°C and transferred into a new bottle with secondary antibodies containing blocking solution for 24 hours at 37°C. Repeat washing as mentioned above was performed and ovaries were subjected to optical clearing into a new bottle containing 5ml CUBIC-reagent-1^41^ and incubated for 2 days. After washing 5x as above, ovaries were transferred into a new bottle containing 1ml 50% of CUBIC reagent-2 and incubated for 1 hour at 37°C. Then ovaries were transferred into a new bottle containing 5ml 100% of CUBIC reagent-2 and incubated for 1 hour at 37°C. Next, the ovaries were transferred to a confocal dish and images were acquired on a Ti2 inverted confocal laser microscope (Nikon) with a 10× objective. Images were collected as Z-stacks with a step size of 3 μm.

Oocytes were counted using markers Tra98 and DDX4. Imaris v 6.4 was used for image processing, and a spot-transformation algorithm was used to identify each oocyte for oocyte counting^42^.

### Neonatal ovary cultures

Neonatal day 2 ovaries were collected and cultured on liquid/air interface using Waymouth’s MB752/1 medium (Gibco) supplemented with pyruvate, penicillin streptomycin and 10% FBS (Wisent) as previously described^43^ for 24 or 48 hours with vehicle (DMSO, Sigma), ATMi (KU55933, 20mM, AdooQ) or Chk2i (10mM, Cayman). After this period, tissue was either fixed for immunohistochemical or histomorphometric analysis of paired ovaries.

### Oocyte collection and spindle staging

Females were superovulated by giving 5 IU of pregnant mare’s serum gonadotropin (ProSpec) followed 48 hours later by 5 IU of human chorionic gonadotropin (Merck) intraperitoneally. Cumulus-oocyte complexes were recovered from oviducts of primed females 14 to 16 hours after hCG injection. Oocytes were denuded from cumulus cells by brief incubation in hyaluronidase (Sigma-Aldrich), fixed in 4 % paraformaldehyde, and processed for immunostaining.

### Comet assay

Ovulated oocytes were used for analysis of DNA integrity by comet assay. To detect DNA breaks, the comet assay was performed under neutral conditions using Single cell Comet Assay Electrophoresis (R&D Systems) following manufacturer’s instructions.

### Fertility tests

*Nemp1*^*−/−*^ and *Nemp1*^*−/−*^*;Chk2*^−/−^ females of 6–8 weeks old were crossed to 8–12 weeks old males with proven fertility. The reproductive activity of females was monitored for up to 6 months from the day of mating by recording the number of litters and pups delivered by each female.

### Real-Time PCR

RNA was extracted from 3 pairs of neonatal ovaries of the same genotype using TRI Reagent (ThermoFisher). Obtained RNA pellet was dissolved in nuclease free water and any residual DNA was removed by brief digestion with RNAse free DNAse (Sigma) and reverse transcription was performed in a volume of 20 μl using Advanced cDNA Synthesis kit (Wisent). Subsequently 1μl of RT was used in a real-time PCR reaction using Advanced SYBR Green PCR Master Mix (Wisent) in CFX96 Touch^™^ Real-Time PCR Detection System (Biorad). Samples were run in triplicates and analysis was done using delta/delta CT method. Beta actin was used to pre-screen the sample quality. However, DDX4 was used for normalisation, as *Nemp1*^*−/−*^ ovaries contain fewer germ cells and targets analysed are known to be expressed by oocytes. Puma: Forward: ATGCCTGCCTCACCTTCATCT/ Reverse: AGCACAGGATTCACAGTCTGGA; Noxa Forward: ACTGTGGTTCTGGCGCAGAT/ Reverse: TTGAGCACACTCGTCCTTCAA; DDX4 Forward: TGGCAGAGCGATTTCTTTTT/ Reverse: CGCTGTATTCAACGTGTGCT.

### Statistical analysis

All the statistical procedures were performed using GraphPad Prism version 10.4.0.621, and data were presented as means ± SEM. For compare, ns of two unpaired samples, significance was determined by student t-test or Mann-Whitney nonparametric test. For two paired samples, significance was determined by paired t-test or Wilcoxon nonparametric test. To analyze the difference between more than two independent groups, statistical analysis was performed by one-way analysis of variance (ANOVA) or two-way analysis of variance (ANOVA) and the significance was determined by Tukey’s multiple comparison test or Kruskal-Wallis for nonparametric data with Dunn’s multiple-comparison test. The results were considered significant if the P value was less than 0.05.

## Supplementary Files

This is a list of supplementary files associated with this preprint. Click to download.

- SupplementaryInformation071825.pdf
